# Manganese acquisition and homeostasis at the host-pathogen interface

**DOI:** 10.3389/fcimb.2013.00091

**Published:** 2013-12-05

**Authors:** John P. Lisher, David P. Giedroc

**Affiliations:** ^1^Graduate Program in Biochemistry, Indiana UniversityBloomington, IN, USA; ^2^Department of Chemistry, Indiana UniversityBloomington, IN, USA

**Keywords:** manganese, ATP-binding cassette, metal transport, homeostasis, nutritional immunity, iron

## Abstract

Pathogenic bacteria acquire transition metals for cell viability and persistence of infection in competition with host nutritional defenses. The human host employs a variety of mechanisms to stress the invading pathogen with both cytotoxic metal ions and oxidative and nitrosative insults while withholding essential transition metals from the bacterium. For example, the S100 family protein calprotectin (CP) found in neutrophils is a calcium-activated chelator of extracellular Mn and Zn and is found in tissue abscesses at sites of infection by *Staphylococcus aureus*. In an adaptive response, bacteria have evolved systems to acquire the metals in the face of this competition while effluxing excess or toxic metals to maintain a bioavailability of transition metals that is consistent with a particular inorganic “fingerprint” under the prevailing conditions. This review highlights recent biological, chemical and structural studies focused on manganese (Mn) acquisition and homeostasis and connects this process to oxidative stress resistance and iron (Fe) availability that operates at the human host-pathogen interface.

## Introduction

The most abundant transition metals in humans are iron (Fe) and zinc (Zn) (for reviews, see Maret, 2010; Hood and Skaar, 2012) and it is generally accepted that a clinical deficiency in host levels of either metal increases the incidence of infectious disease and mortality (Haider and Bhutta, 2009; Kumar and Choudhry, 2010; Lassi et al., 2010). These deficiencies reduce the ability of the host to utilize these metals to restrict bacterial growth. In the case of Fe, many peroxide- and nitrous oxide-generating enzymes are iron-dependent, and a limitation of iron would compromise this aspect of innate immunity against bacterial pathogens (Kumar and Choudhry, 2010). Zinc deficiency compromises function of the human immune system (Kitamura et al., 2006) and the ability of the host to induce zinc-mediated cellular toxicity as a means to control bacterial infections (for a review, see Stafford et al., 2013); as a result, this condition is associated with an increased incidence of serious infectious disease (Lassi et al., 2010). In contrast, although manganese (Mn) overload is connected to neurological dysfunction (for a review, see Rivera-Mancía et al., 2011) there is not as yet, strong support for the idea that host Mn(II) sufficiency is in any way coupled to the incidence or severity of infectious disease.

There is now, however, emerging evidence that the invading microbe utilizes Mn as a key micronutrient to resist the effects of host-mediated oxidative stress and thus plays a significant role in adaptation of pathogenic bacteria to the human host. This review summarizes recent work in the area of “Mn-centric” nutritional immunity (Weinberg, 1975) placed in the context of the inorganic physiology of the cell and the “fight over metals” implied by recent studies of Mn speciation and chemistries of low molecular weight (LMW) Mn complexes (McNaughton et al., 2010; Barnese et al., 2012; Sharma et al., 2013), and the structures and metal binding affinities of the bacterial high affinity import systems for Mn relative to the extracellular antibacterial protein calprotectin (Corbin et al., 2008; Damo et al., 2013; Hayden et al., 2013). Calprotectin possesses functional properties consistent with that of an extracellular Mn chelator that withholds this metal from the invading pathogen (Kehl-Fie et al., 2013) and is thus formally analogous to siderochalins that capture Fe-siderophores synthesized by the pathogen itself (Flo et al., 2004; Sia et al., 2013). Finally, recent insights into the coordinate regulation and crosstalk that govern intracellular Mn vs. Fe and Mn vs. Zn bioavailability will also be discussed.

## Inorganic chemistry of the cell: total metal and cellular metal speciation

**Figure 1:**
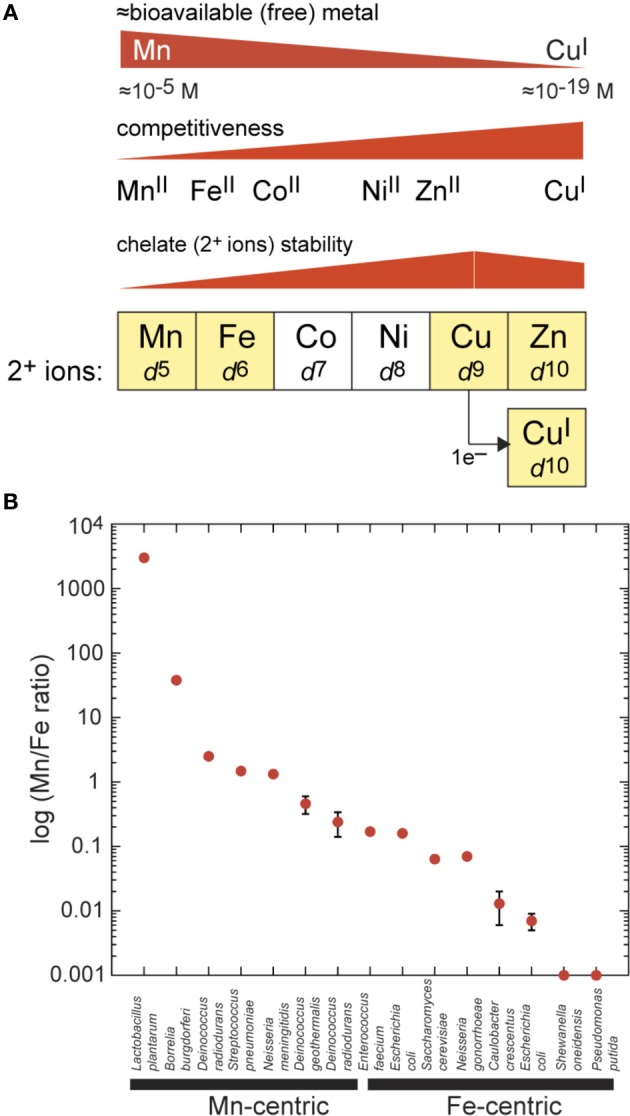
**Inorganic chemistry of the cell. (A)** Biologically important first-row transition metals extracted from the periodic table from Mn to Zn and with outer (3*d*) shell electronic configurations (*d*^5^–*d*^10^) as indicated. Zn(II) and Cu(I) both have filled *d*-shells. Coordination number (CN) preferences move from Mn to Cu(I) from high [CN = 6 for Mn(II)] to low [CN = 2–4 for Cu(I)], which tracks with increasing thiophilicity and polarizability of the metal. The approximate trend in metal(II) complex stability for a mixed N/O ligand donor set is indicated for each metal (Fraústo da Silva and Williams, 2001); this trend is inversely related to competitiveness in a cellular environment which itself is inversely related to “bioavailability” of each metal in the cell. Bioavailability is very roughly based on reported metal sensor affinities for their cognate metal and is not a direct measure (Reyes-Caballero et al., 2011). **(B)** Total cell-associated log (Mn:Fe ratio) plotted for individual bacteria as measured by ICP-MS. The term Mn-centric is operationally defined here as those organisms for which Mn:Fe ≥0.2, or the lower limit obtained for *Deinococcus radiodurans* under extremely Mn-depleted growth conditions (Daly et al., 2004). Fe-centric refers to those organisms for which Mn:Fe ≤0.2. Most of these analyses were obtained for exponentially growing cells on a rich growth medium (given in parentheses) with no added Mn(II), unless otherwise indicated. These measurements were taken for *Lactobacillus plantarum* (All Purpose Tween, APT) (Posey and Gherardini, 2000), *Borrelia burgdorferi* [Barbour-Stoenner-Kelly media (BSK-II) or Modified serum-free media with Exyte (SF-E)] (Posey and Gherardini, 2000), *Deinococcus radiodurans* (Defined Minimal media + 2.5 μM Mn) (Daly et al., 2004), *Streptococcus pneumoniae* (Brain Heart Infusion, BHI) (Jacobsen et al., 2011), *Neisseria meningitidis* (Gonococcal Broth, GCB) (Veyrier et al., 2011), *Deinococcus geothermalis* (Tryptone Glucose Yeast extract media, TGY) (Daly et al., 2004), *Deinococcus radiodurans* (TGY) (Daly et al., 2004), *Enterococcus faecium* (TGY) (Daly et al., 2004), *Escherichia coli* (Luria Broth, LB) (Outten and O'Halloran, 2001), *Saccharomyces cerevisiae* (Yeast extract Peptone Dextrose media, YPD) (Rosenfeld and Culotta, 2012), *Neisseria gonorrhoeae* (GCB) (Veyrier et al., 2011), *Caulobacter crescentus* (Peptone Yeast Extract media, PYE) (Hughes et al., 2013), *Escherichia coli* (TGY) (Daly et al., 2004), *Shewanella oneidensis* (TGY; Mn/Fe 0.0005 ± 0.00004) (Daly et al., 2004), and *Pseudomonas putida* (TGY; <0.001) (Daly et al., 2004).

Six first-row 3*d*-block elements extending from manganese to zinc are essential micronutrients that function as inorganic cofactors in up to 25% of all proteins in cells (Figure 1A) (Waldron and Robinson, 2009). Mn, Fe, Cu and Zn are ubiquitous in biology (Maret, 2010) while cobalt (Co) and nickel (Ni) play more specialized roles in methyl transfer chemistry (Gherasim et al., 2013) and as a cofactor for a limited number of Ni(II)-containing metalloenzymes (Kaluarachchi et al., 2010), respectively. Zn is unique among these metals in that it is redox-inert and thus stable in the 2+ oxidation state. As such, Zn functions as nature's principal Lewis acid catalyst, where it activates a substrate for catalysis. Zn is a cofactor in a wide range of hydrolytic enzymes, constitutes the largest fraction of metalloproteins in the cell (Maret, 2001) and stabilizes protein structure in the reducing environment of the cytoplasm. Fe readily accesses Fe(II) and Fe(III) oxidation states in 2Fe-2S and 4Fe-4S iron-sulfur proteins involved in electron transfer, with higher valence Fe(IV)-oxo species in both heme and non-heme Fe-containing enzymes responsible for significant oxygen insertion and oxygen activation chemistry (Andrews et al., 2003). Fe is also the obligate cofactor for Fe-superoxide dismutase (Fe-SOD) and nearly all ribonucleotide reductases, although more recently, di-Mn-containing variants have been discovered and characterized (for a review, see Cotruvo and Stubbe, 2012).

Mn, like Zn, is a Lewis acid cofactor in a number of hydrolytic enzymes, e.g., protein phosphatases, and in key enzymes of intermediary metabolism, but is redox-active [to Mn(III), Mn(IV) and Mn(V)] and is most strongly linked to oxidative stress resistance mediated by Mn superoxide dismutase (Mn-SOD) (Culotta et al., 2006) and non-heme di-Mn catalases (Whittaker, 2012). As discussed below, simple small molecule Mn(II)-complexes, unique to Mn(II), may have substantial antioxidant activity inside cells (Barnese et al., 2008, 2012). Finally, the bacterial requirement for intracellular Cu, outside of the photosynthetic bacteria, is generally accepted to be low (Waldron et al., 2009); as a result, there is emerging evidence that the human host harnesses the cytotoxic power of Cu to kill invading bacterial pathogens (White et al., 2009; Rowland and Niederweis, 2012; Samanovic et al., 2012); this need not, however, be the case for all microbial pathogens (Raja et al., 2013).

**Figure 2:**
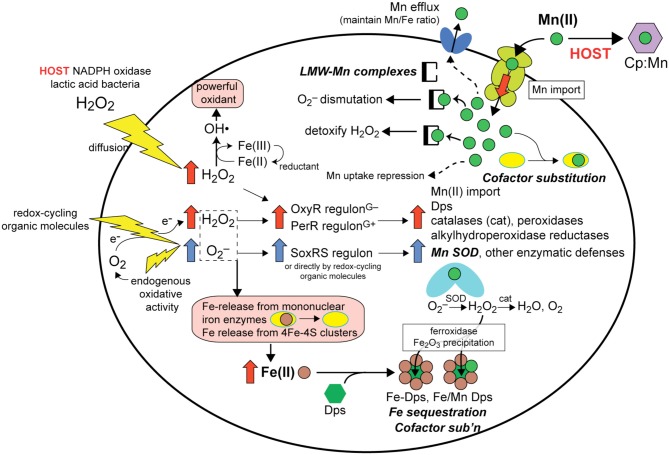
**Overview of the responses available to a hypothetical bacterial pathogen exposed to host derived exogenous or endogenous ROS (O_2_^−^, redox-cycling organic molecules and H_2_O_2_) and how upregulation of the OxyR (Gram-negative)/PerR (Gram-positive) and SoxRS regulons (Gu and Imlay, 2011) leads to protection against ROS-mediated damage (for a review see Imlay, 2013)**. Here, we focus on the antioxidant effects that derive from upregulated Mn(II) import. (Note: not all processes are known to occur in all cells). The primary molecular targets of ROS in Fe-centric bacteria are Fe (*rust*-filled circles)-release from mononuclear Fe enzymes and from 4Fe-4S clusters (*pink* boxes, lower left), which must be avoided to due to the autocatalytic formation of the freely diffusible hydroxyl radical (OH•). (*pink*, upper left). Four cellular responses involving changes in Mn or Fe speciation as a result of ROS are schematized and highlighted (*bold-face*). (1) Fe sequestration: Fe(II) is scavenged by Dps (*green* triangle) to form Fe-Dps which oxidizes Fe to insoluble Fe oxide using O_2_ or H_2_O_2_ as an oxidant and limiting catalytic formation of OH• (*bottom, middle*). As a result of PerR/OxyR regulatory responses to ROS, increased expression of the Mn (*upper right*; ABC transporter schematic shown) importer lead to increased intracellular Mn(II) (*green circles*) that (2) increases the concentration of LMW-Mn complexes that function to dismutate O_2_^−^ and possible to detoxify H_2_O_2_ (*upper middle*) (see Figure 4), (3) permits cofactor substitution of mononuclear Fe-containing enzymes (*yellow* ellipses; *right*) and perhaps of Dps (*lower right*) with Mn, and (4) enhances the metallation and increased activity of Mn SOD (*middle, right*). Hosts attempt to limit these responses through direct competition of the Mn(II) transporter with host-encoded calprotectin (CP; *purple* hexagon) for Mn (*upper right*) (see text for details). Increased intracellular Mn(II) will ultimately be sensed by the Mn-activated repressor (DtxR, PsaR, etc.) to repress uptake, while excess Mn(II) will be effluxed from the cell by a limited number of organisms (Rosch et al., 2009; Veyrier et al., 2011) in order to bring the Mn/Fe ratio back into balance (Veyrier et al., 2011).

The inorganic “fingerprint” of unstressed cells is defined as the *total* concentration of all cell-associated metals summed over all cellular fractions (membrane, cytoplasm, periplasm, etc.). This is generally expressed in nmol or ng of each metal per mg total protein and is readily measured by inductively coupled plasma mass spectrometry (ICP-MS) of acid-solubilized cells. A remarkable aspect of the inorganic fingerprint relevant to this discussion is the fact that the total Mn:Fe ratio varies by over *seven orders* of magnitude when various single-celled organisms are compared with one another (Figure 1B). If *Escherichia coli* is taken as a typical bacterium, then Zn is as abundant as Fe, with Mn and Cu present at ≈10-fold lower concentration, and Ni and Co about 10–50 fold lower still (Outten and O'Halloran, 2001; Maret, 2010), giving a Mn:Fe ratio of ≈0.1. This fingerprint tends to characterize “Fe-centric” bacteria, like *E. coli* and the yeast *Saccharomyces cerevisiae* (Outten and O'Halloran, 2001; Rosenfeld and Culotta, 2012) with some Fe-centric species accumulating only vanishingly small amounts of Mn (Mn:Fe ratio ≤0.001) (Figure 1B) (Daly et al., 2004). On the other hand, for some Gram-positive bacteria, total cell-associated Mn levels are on par with that of Zn, with Fe levels correspondingly lower, resulting in a Mn:Fe ratio of ≥1 for these Mn-centric organisms (Figure 1B) (Daly et al., 2004; Jacobsen et al., 2011; Veyrier et al., 2011). These bacteria include the lactic acid bacteria *Streptococcus pneumoniae* and *Lactobacillus plantarum*, the pathogen *Neisseria meningitidis*, and the UV-resistant *Deinococcus radiodurans*. In *Deinococcus radiodurans*, Mn is known to play a direct role in protecting this organism from the effects of extreme γ-radiation (Daly et al., 2004). As a general rule, lactic acid bacteria tend to have less in the way of an intracellular Fe requirement relative to *E. coli*, and this may be a consequence of the unusual lifestyle of these organisms which lack a respiratory chain and yet generate millimolar hydrogen peroxide (H_2_O_2_) when grown in the presence of oxygen (Archibald and Fridovich, 1981b; Ramos-Montañez et al., 2010). This would play havoc with an Fe-centric bacterium and in fact is used by lactobacilli to kill other bacteria in the microbial community (see Figure 2 below).

It is important to emphasize that the total metal content of an organism does not dictate the relative concentrations of *free* metal, which we define here as the fraction of total metal that is rapidly exchangeable with LMW chelates or cellular metabolites and is thus bioavailable (Figure 1A). This bioavailable metal tends to track with the left-to-right arrangement of the *d*-block elements in the periodic table, which is inversely related to metal competitiveness within a cellular environment. Metal competitiveness, in turn, is roughly governed by the intrinsic chelate stability, which provides a measure of the equilibrium ability of one metal to displace another metal from an enzyme active site, for example (Fraústo da Silva and Williams, 2001) (Figure 1A). Both Zn(II) and Cu(I) are highly competitive *d*^10^ metals and thus will outcompete all other divalent transition metals in the first row, in particular Mn(II) and Fe(II), if left unregulated (for reviews, see Waldron and Robinson, 2009; Reyes-Caballero et al., 2011). In short, there must be a cellular overcapacity to chelate Zn(II) in order to keep this highly competitive metal in check (Outten and O'Halloran, 2001) (Figure 1A). In contrast, for Mn(II) (McNaughton et al., 2010; Sharma et al., 2013) and perhaps Fe(II) in *E. coli* (Imlay, 2008) a significant fraction of the total cell-associated metal is found in rapid equilibrium with a chelatable pool of LMW metabolite-metal complexes, e.g., amino acids, nucleosides, nucleotides, orthophosphate, citrate, and carbonate. This has significant implications for Mn(II) and microbial pathogenesis, as discussed below.

## Transition metal homeostasis and the fight over metals

The cellular bioavailability of transition metals is governed by continuous cycles of adaptation and recovery to changes in extracellular metal availability, e.g., that which might occur along an infection axis. This process is termed transition metal homeostasis. Metal homeostasis systems maintain both total and bioavailable metal concentrations to maximize cell viability under the prevailing extracellular milieu. This process is orchestrated by a panel of metal sensor proteins that regulate the transcription of genes encoding metal uptake, metal efflux and metal sequestration proteins. Metal sensor proteins are typically repressors whose DNA operator-promoter binding or transcription activation activity is reversibly modulated by the binding of one or more cognate (-like) metal ions to the exclusion of all others (Giedroc and Arunkumar, 2007; Ma et al., 2009). This regulatory process has a tremendous impact on the survival and pathogenesis of microbial pathogens (Andreini et al., 2008; Botella et al., 2012). For example, Cu(I) and Zn(II) compete with native metals leading to mismetallation of metalloenzymes with more weakly bound metals and loss of function (Aguirre and Culotta, 2012; Botella et al., 2012; Cotruvo and Stubbe, 2012), while ROS and Fe cause deleterious reactions leading to oxidative damage of proteins, DNA and lipids (Imlay, 2013) (see Figure 2; discussed more fully below).

For bacterial pathogens, proper metallation of critical proteins also competes against host defenses that have evolved to limit or sequester these required micronutrients to quell a bacterial infection, thus creating a “fight over metals” (for a review, see Hood and Skaar, 2012). Furthermore, this fight is intermingled with ongoing global stress mediated by reactive oxygen species (ROS) (Imlay, 2013), reactive chlorine species (HOCl) (Gray et al., 2013) and/or reactive nitrogen species (RNS) (Stern et al., 2013) to which the pathogen must adapt and ultimately exploit (Hoffmann et al., 2006). The ability of the host immune system to sequester transition metals is an important aspect of nutritional immunity (Kehl-Fie and Skaar, 2010). Although long recognized for Fe limitation (Weinberg, 1974, 1975; Forbes and Gros, 2001; Flo et al., 2004; Skaar, 2010; Hammer and Skaar, 2012), it is now established that both Zn and Mn availability are actively limited by the host as well, through the extracellular action of calprotectin (CP) (Corbin et al., 2008) and perhaps other molecules. Indeed, the host strategy of limiting Mn, in particular, results in sensitization to oxidative stress (Anjem et al., 2009; Ogunniyi et al., 2010; Kehl-Fie et al., 2011) which limits the ability of Mn(II) to function as an antioxidant as discussed below.

## Manganese as an antioxidant micronutrient

**Figure 3:**
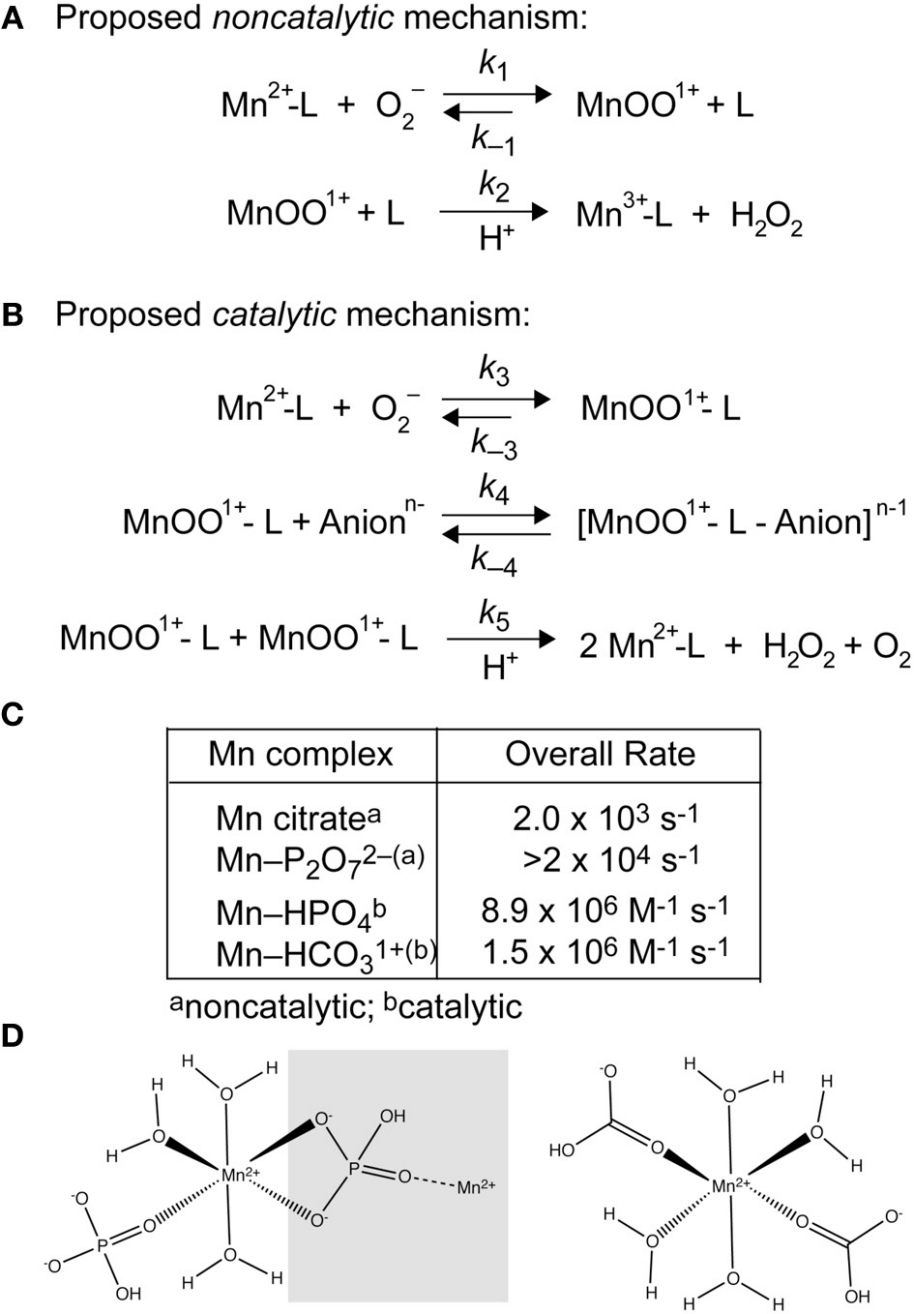
**Superoxide disproportionation by LMW-Mn complexes (Barnese et al., 2012). (A)** Plausible noncatalytic mechanism of the reaction of O_2_^−^ with [Mn(II)-P_2_O_7_]^2−^ and Mn(II)-citrate. L, pyrophosphate or citrate. **(B)** Proposed catalytic mechanism of the reaction of O_2_^−^ with [Mn(II)-HPO_4_] and [Mn(II)-HCO_3_]^+^ leading to the catalytic disproportionation of O_2_^−^. L, phosphate or carbonate; Anion^*n*−^ represents an additional bound anion to the form the intermediate. **(C)** Overall rate constants for the non-catalytic and catalytic disproportionation of O_2_^−^, the latter of which incorporates the MnOO+ dependence of *k*_5_. Rate law simulations reveal that 91 μM MnHPO_4_ (165 μM Mn(II), 5 mM phosphate), and 25 μM MnHCO_3_^+^ (formed by 36 μM Mn(II) and 5 mM carbonate) gives rise to a steady-state [O_2_^−^] (3 μM) identical to 1 μM CuZn-SOD following a 25 μM burst of superoxide (Barnese et al., 2012). Thus, intracellular Mn(II) in the ≈100 μM range is expected to be sufficient to resist the effects of superoxide stress, as found previously in yeast; these studies also physically document the presence of MnHPO_4_ species in whole cells (McNaughton et al., 2010). The same may well be true for manganese-centric bacterial pathogens vs. iron-centric *E. coli* (Aguirre et al., 2013; Sharma et al., 2013) (see Figure 1B). **(D)** Chemical structures of “layered” Mn-HPO_4_, where the *gray* box encompasses the next layer in the crystal lattice (*left*) (Krishnamohan Sharma et al., 2003) and a calculated model of frozen neutral [Mn(HCO_3_)_2_] from ENDOR studies (*right*) (Potapov and Goldfarb, 2008).

Manganese as a micronutrient is critical to the viability and virulence of many Gram-positive and Gram-negative bacterial pathogens (Tseng et al., 2001; Johnston et al., 2004; Ogunniyi et al., 2010; Kehl-Fie et al., 2011; Wichgers Schreur et al., 2011; Perry et al., 2012). In these bacteria, deletion of either the manganese import system(s) or the associated Mn(II)-specific metal sensor protein compromises virulence and/or viability often through sensitizing the bacteria to various ROS, e.g., superoxide anion radical (denoted here as O_2_^−^) or hydrogen peroxide (H_2_O_2_) (Johnston et al., 2006; Abrantes et al., 2013) (Figure 2). Major consequences of O_2_^−^ and H_2_O_2_ toxicity in cells is the autocatalytic production of the highly damaging hydroxyl radical, OH•, via the Fenton reaction from these partially reduced forms of O_2_, and the oxidative attack and dissociation of solvent-exposed Fe(II) atoms from enzymes harboring mononuclear Fe and 4Fe-4S cluster cofactors (Figure 2). Here, a solvent-exposed Fe(II) atom will allow for direct coordination of H_2_O_2_ leading to a local generation of OH• and protein oxidation and Fe(III) dissociation, essentially analogous to that which occurs in the peroxide sensor Fe-PerR (Lee and Helmann, 2006) (Figure 3). As such, all bacteria encode regulatory strategies to quickly respond to various ROS. In *E. coli*, low (μM) H_2_O_2_ induces the OxyR regulon which includes genes encoding a manganese import pump MntH and the DNA binding iron-scavenging protein Dps (Zheng et al., 2001), as well as catalases and peroxidases capable of reducing ROS or organic peroxides (Figure 3). In Gram-positive bacteria, PerR carries out essentially the same regulatory role as OxyR. Redox-cycling organic molecules (Gu and Imlay, 2011) and perhaps O_2_^−^ itself induce the SoxRS regulon, a major component of which is Mn-superoxide dismutase (SOD).

As a result of ROS stress, four major cellular adaptations result that collectively limit pro-oxidant Fe availability and upregulate Mn import to harness the antioxidant properties of Mn(II). First, increased Mn(II) availability allows for increased metallation of Mn-SOD which efficiently catalyzes the dismutation of the superoxide anion radical to H_2_O_2_ and O_2_, the former of which is cleared by catalase and related peroxidases which are also induced under these conditions (May and Dennis, 1989; Wintjens et al., 2004) (Figure 2). However, as early as 1981, a series of studies established that LMW manganese-metabolite complexes from extracts of *Lactobacillius plantarum* (see Figure 1B) were capable of scavenging superoxide from solution (Archibald and Fridovich, 1981a,b) (Figure 2). A number of diverse bacterial species (Inaoka et al., 1999; Tseng et al., 2001; Al-Maghrebi et al., 2002; Daly et al., 2010; Kehl-Fie et al., 2011) and the baker's yeast *Saccharomyces cerevisiae* (Chang and Kosman, 1989; McNaughton et al., 2010; Reddi and Culotta, 2011) are now known to posses this activity as well, although in most cases it is supplemented by SOD enzyme-catalyzed superoxide dismutation, with the prominent exceptions of *L. plantarum* and *Neisseria gonorrhoeae* (Tseng et al., 2001).

Simple Mn-phosphate (P_i_) and Mn-carbonate complexes are efficient catalysts of superoxide disproportionation and the chemical mechanism of this reaction has recently been investigated in detail (Barnese et al., 2008, 2012) (Figure 3). This catalysis occurs at physiologically relevant rates and metabolite concentrations and may well-explain studies that connect oxidative stress resistance to phosphate accumulation and changes in phosphate metabolism (Tseng et al., 2001; Jensen et al., 2003; McNaughton et al., 2010; Rosenfeld et al., 2010; Wu et al., 2010). Additionally, manganese carbonate complexes have been shown to catalyze the decomposition of H_2_O_2_ (Figure 2) suggesting that other small molecule Mn-complexes can potentially function downstream of superoxide, although this reaction has not been thoroughly investigated (Stadtman et al., 1990; Liochev and Fridovich, 2004). Recent findings in *S. aureus* support the presence of manganese-dependent, SOD-*independent* mechanisms to effectively scavenge superoxide (Kehl-Fie et al., 2011).

In addition to the chemical clearance of ROS mediated by LMW-Mn complexes, cofactor substitution (see Figure 2) of Fe(II) for Mn(II) in selected mononuclear iron enzymes has recently been proposed to protect these enzymes from redox chemistry at the Fe active site that accompanies H_2_O_2_ stress (Imlay, 2013) (Figure 2). Indeed, many mononuclear Fe enzymes are reversibly inactivated by H_2_O_2_, and continued exposure leads to irreversible inactivation (Sobota and Imlay, 2011; Anjem and Imlay, 2012). This Fe(II)-for-Mn(II) substitution is facilitated by the generally weak binding (rapid dissociation) of these two metals to enzymes (see Figure 1A) and is predicted to function well in enzymes that employ Fe(II) in Lewis acid catalysis, given the similar coordination preferences of Fe(II) and Mn(II); however, this process is projected to fail when the Fe(II) atom needs to undergo a change in oxidation state, given the very different redox potentials of these two metals (Cotruvo and Stubbe, 2012). In any case, Mn is known to protect these enzymes from inactivation *in vitro* and *in vivo* where both manganese import and iron sequestration were required for protection (Anjem and Imlay, 2012) (Figure 2). These studies reveal that *E. coli* is capable of shifting from a metabolism based on Fe(II) to one based on Mn(II) in order to protect key enzymes from inactivation by ROS. Another example of this type of cofactor replacement is the iron sequestration protein, Dps, in *Kineococcus radiotolerans* (Figure 2). Dps is a binuclear Fe-containing ferroxidase that binds Fe and precipitates iron oxide inside of the Dps dodecamer; however, the *K. radiotolerans* enzyme is also active as a mixed metal Mn-Fe enzyme, further evidence for the protective role of Mn(II) via cofactor substitution in the oxidative stress response (Ardini et al., 2013).

Thus, Mn(II) functions as an antioxidant through a combination of enzymatic degradation of oxidants by Mn-SOD and other Mn(II)-containing enzymes, nonenzymatic degradation of oxidants by LMW-Mn complexes, and metalloenzyme cofactor substitution to prevent Fe-induced peroxide chemistry and subsequent enzyme inactivation (Figure 2). The extent to which each process contributes likely varies from organism to organism and will be dependent on the prevailing microenvironment. However, a metabolism capable of utilizing Mn(II) and not absolutely dependent on Fe may well-represent a general strategy that nature has evolved to develop robust viability in the presence of significant or chronic ROS. The causative agent of Lyme disease, *Borrelia burgdorferi*, which completely lacks an Fe requirement and as a result is characterized by a very high Mn:Fe ratio (Figure 1B), may represent an extreme example of this evolutionary adaptation that protects the organism from host-mediated ROS (Posey and Gherardini, 2000; Aguirre et al., 2013).

## Structural studies of bacterial manganese import systems

The ability of a bacterial pathogen to obtain sufficient Mn(II) is critically important for pathogenesis and as such, Mn(II)-dependent metal sensor proteins control the expression of operons that encode additional virulence factors unrelated to the acquisition of Mn(II) (Gold et al., 2001; Johnston et al., 2006; Rolerson et al., 2006; Hendriksen et al., 2009). This suggests that Mn(II) limitation may well be a generic signal that poises the invading pathogen to quickly adapt to a wide range of host immune defenses. Unlike the case for Fe siderophores, there is no known LMW, high affinity chelator that is secreted by bacteria to scavenge Mn from the environment. As a result, the capture and transport of manganese into the cell is facilitated directly by manganese import systems, which include MntH, a NRAMP1 (natural resistance-associated macrophage protein 1)-family transporter and the Mn/Fe/Zn-specific cluster A-I ABC (ATP-Binding Cassette) transporters (Dintilhac and Calverys, 1997; Papp-Wallace and Maguire, 2006; Berntsson et al., 2010).

**Figure 4:**
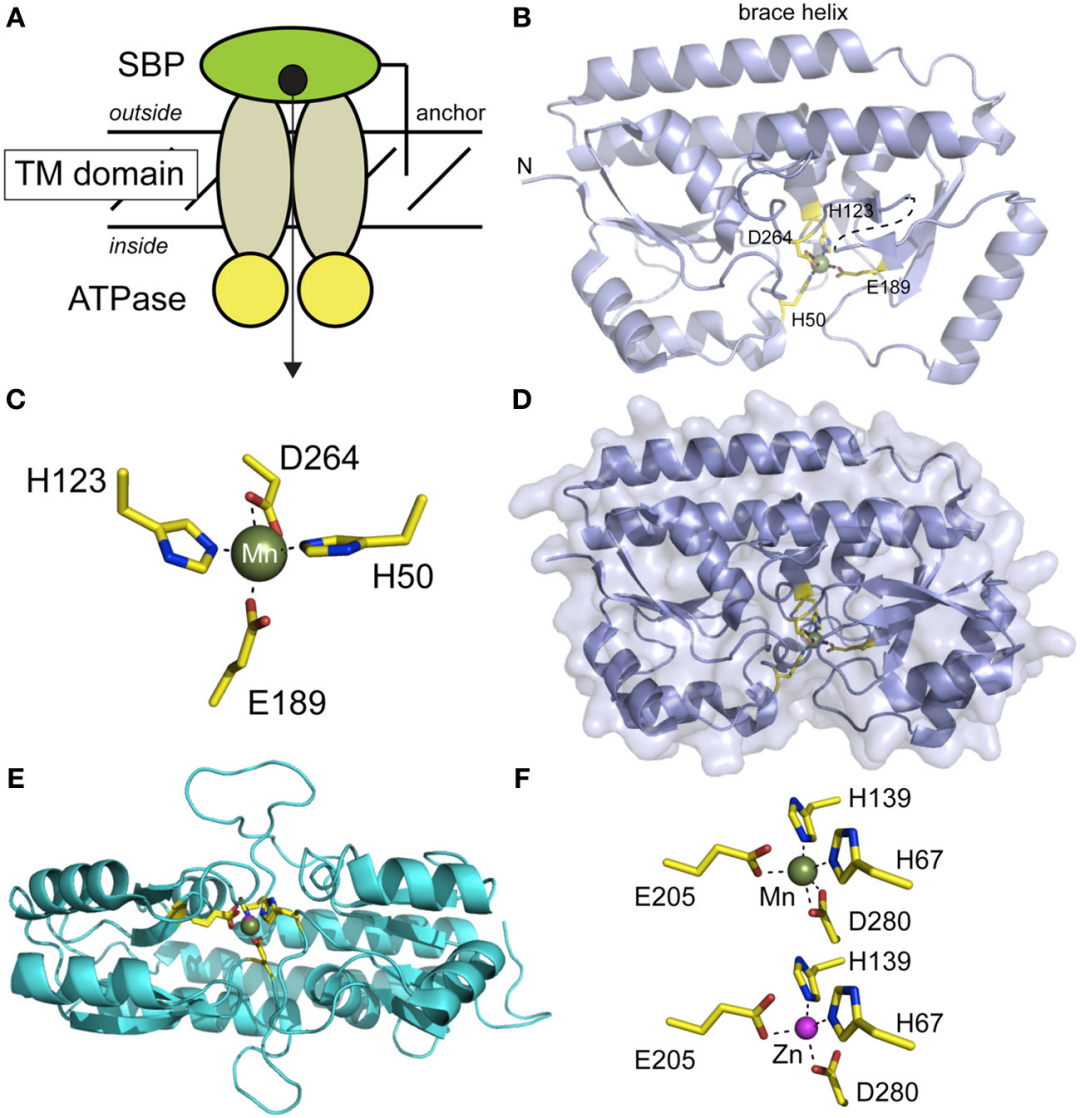
**Structural studies of Mn-specific solute binding proteins (SBPs) from *S. aureus* MntC (panels B-D) (Gribenko et al., 2013) and *S. pneumoniae* PsaA (panels E,F) (Lawrence et al., 1998; McDevitt et al., 2011). (A)** Cartoon representation of a canonical ABC transporter, with the subunits and the direction of transport labeled. **(B)** Ribbon representation of the structure of Mn-bound MntC (pdb code 4K3V), with the four Mn(II)-coordinating ligands highlighted and shown in *stick*. This orientation is similar to that implied by the cartoon in panel **(A)**. **(C)** First coordination sphere of the Mn(II) complex in MntC; this orientation differs from that in panel B in order to highlight the distorted trigonal bipyramidal Mn(II) coordination geometry. E189 tends toward monodentate ligation, while D264 is bidentate. **(D)** Surface representation of MntC in the same orientation as in panel B revealing that bound Mn(II) is buried from solvent. **(E)** Ribbon representation of a global superposition of PsaA in the Mn(II)-bound (3ZTT) and Zn(II)-bound (1PSZ) states, with metal ligands shown in *stick*. This orientation is from the docking surface that would interact with the transmembrane subunits (see panel **A**). **(F)** Coordination complexes of PsaA bound to Mn(II) (*top*) and Zn(II) (*bottom*) revealing that the same four protein-derived ligands are used to coordinate both cognate and noncognate metals. The Mn(II) complex is very similar to that observed for MntC, with E205 tending toward monodentate ligation (*r*O_e2••_Mn = 2.55 Å). In contrast, the Zn(II) complex tends toward tetrahedral coordination with only one oxygen atom of each carboxylate group sufficiently close to directly coordinate the Zn(II). The overlay of these two chelates is shown in panel **(E)**.

Although structural studies of bacterial NRAMP1 Mn/Fe-transporters are limited, some molecular-level insights are available from extensive modeling studies for this large family of proteins (Cellier, 2012). Limited structural information is available for intact multisubunit cluster A-I ABC transporters as well, although the crystallographic structures of the distantly related bacterial cobalamin transporter BtuC_2_D_2_F (Locher et al., 2002; Korkhov et al., 2012) and the molybdate transporter (ModB_2_C_2_A) are known to modest resolution (Hollenstein et al., 2007). Significant structural data are, however, available for component metal-binding subunits of cluster A-I ABC transporters, termed the solute-binding proteins (SBPs) (Figure 4A). These include a number of Mn- and Zn-specific SBPs (Lawrence et al., 1998; Lee et al., 1999, 2002; Banerjee et al., 2003; Rukhman et al., 2005; Chandra et al., 2007; Li and Jogl, 2007; McDevitt et al., 2011; Zheng et al., 2011; Gribenko et al., 2013). It is generally believed that the SBP defines the metal specificity of transport, although precisely how this is accomplished is currently unknown. In addition, Zn is generally a poor substrate and in some cases, a competitive inhibitor, of Mn-specific ABC transporters (DeWitt et al., 2007) despite forming a very similar coordination complex to that of Mn(II) (see below).

The first of the cluster A-I SBPs (Berntsson et al., 2010) to be structurally characterized was PsaA from *Streptococcus pneumoniae* (Lawrence et al., 1998), solved as the Zn(II)-complex to 2.0 Å resolution, and lacking the N-terminal LXXC motif required to anchor PsaA to the lipid membrane. We use the recently determined structure of Mn(II)-bound *S. aureus* MntC to illustrate the fold of this subfamily of SBPs, described as a “Venus fly trap” containing two homologous mixed (βα_4_ sandwich domains linked via a ≈30 amino acid helix that resembles a backbone brace for this two-domain molecule (Figure 4B). The metal binding site is located in a deep cleft between the two domains of MntC, and metal ligands are contributed by both domains in roughly homologous positions (H50, H123 in the N-terminal domain; E189, D264 in the C-terminal domain). The structure of the apo-state of MntC is unknown, but structural studies of ligand-free *Treponema* TroA (Lee et al., 2002) and the SBP specific for vitamin B_12_ (BtuF) reveal essentially closed, metal bound-like structures, with BtuF indicative of a slightly more open and conformationally dynamic structure that collapses around the Co(II)-ligand complex (Karpowich et al., 2003). The Mn(II) in MntC is bound to four protein-derived ligands in what is best described as a pentacoordinate distorted trigonal bipyramidal coordination geometry (Figure 4C) that is completely shielded from solvent (Figure 4D). The structure of the Mn(II)-bound form of PsaA has also recently been solved and compares very favorably with that of Mn-MntC (Figures 4E,F) (McDevitt et al., 2011). While the overall structure is virtually identical with that of Zn(II)-PsaA complex determined earlier (see Figure 4E for an overlay) (Lawrence et al., 1998), there are subtle differences in the metal coordination site, with the Zn(II) complex tending toward distorted tetrahedral as a result of monodentate coordination by each of the two carboxylate ligands (E205, D280) (Figure 4F). These trends in metal coordination geometry of Mn(II) vs. Zn(II) are consistent with expectations (Dudev and Lim, 2013), although the resolution of the structures precludes a stronger conclusion on this point. It is important to point out that some Zn-specific SBPs, e.g., *E. coli* ZnuA, lack the bidentate Asp ligand of Mn-specific SBPs, e.g., D280 in Figure 4F, and recruit a solvent molecule to complete the tetrahedral coordination complex using the other three Mn-SBP ligands (Chandra et al., 2007). This may well have strong implications for metal specificity and the forward rate of cognate or native metal transport across the membrane. A recent paper provides new insights on molecular basis of functional discrimination of cognate Mn(II) vs. non-cognate Zn(II) by *S. pneumoniae* PsaA (Couñago et al., 2013).

The structures of other Mn(II)-specific cluster A-I SBPs have been reported including those from distant bacterial phyla such as cyanobacteria (Rukhman et al., 2005) and the spirochaete *Treponema*, the causative agent of syphilis (Lee et al., 1999). Each structure shares the same MntC/PsaA fold revealing that the (βα_4_ sandwich two-domain structure is evolutionarily conserved and is utilized for transition metal transport in both Gram-negative and Gram-positive bacteria and in nonpathogenic and pathogenic bacteria alike. Given the ubiquity of these proteins on the “outside” of Gram-positive organisms, they have been targeted for use in commercial vaccines. For example, improved serotype coverage and clearance of *Streptococcus pneumoniae* has been obtained with a vaccine containing adjuvant-conjugated PsaA, PiuA and PiaA, the latter two of which are involved in Fe-uptake in this organism (Brown et al., 2001; Whaley et al., 2010).

**Figure 5:**
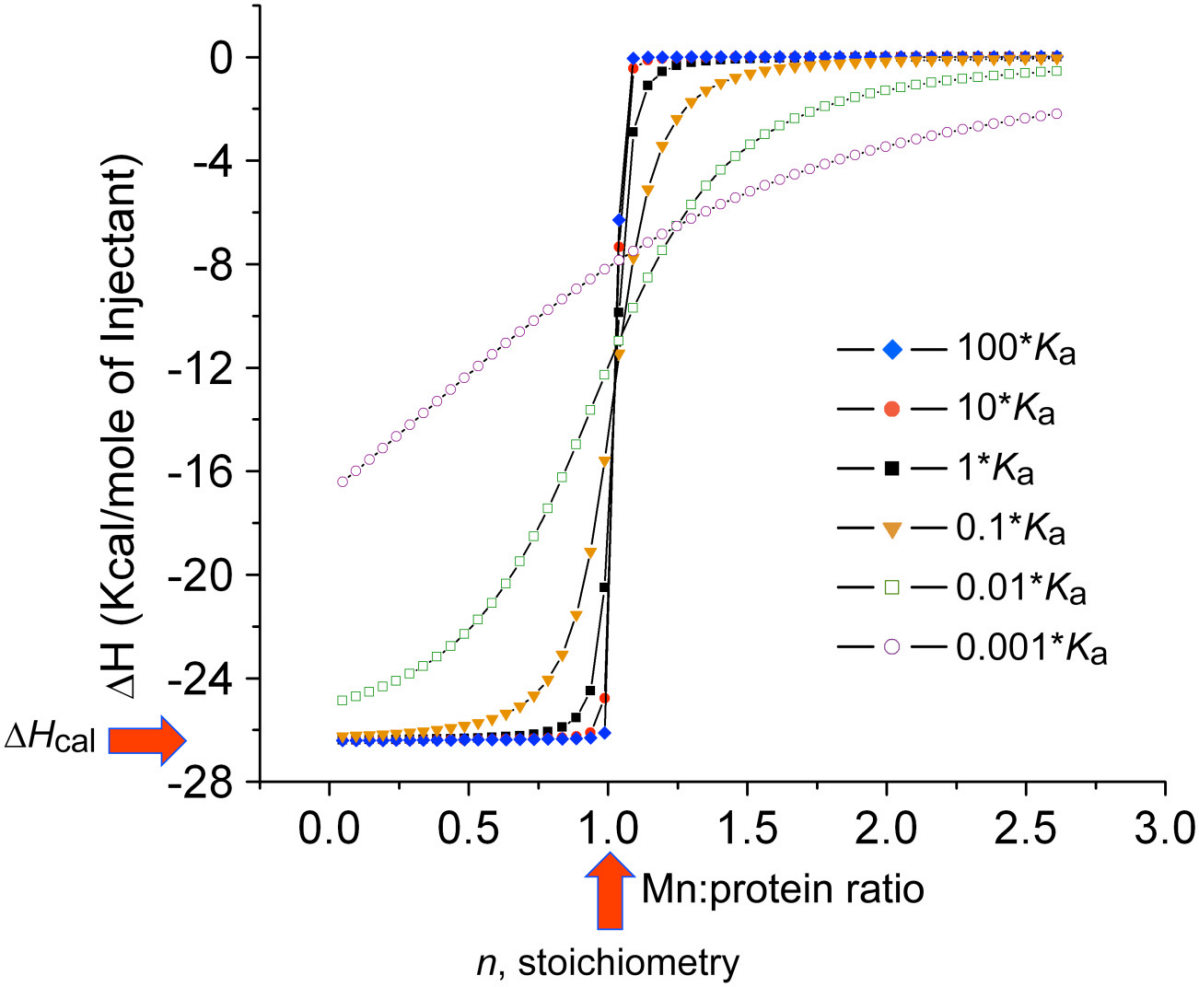
**Simulated binding curves for ITC thermograms using the reported thermodynamics and injection volumes of the MnCl_2_ binding to the SΔ2 mutant of CP (Damo et al., 2013)**. The simulated curve that corresponds to the reported *K*_*d*_ of 5.8 nM (at 10 μM total CP heterodimer), *n* = 1.0 (*red* vertical arrow) and Δ*H*_cal_ = 26.2 kcal mol^−1^ (*red* horizontal arrow) was converted to *K*_a_ (1.7 × 10^8^M^−1^) to create the isotherm labeled 1•*K*_a_ (*black* squares). Other simulated curves are shown for *K*_a_ for 10– (10 * *K*_a_) and 100-fold (100 * *K*_a_) higher *K*_a_, and 10– (0.1 * *K*_a_), 100– (0.01 * *K*_a_) and 1000-fold (0.001 * *K*_a_) lower *K*_a_. These simulations reveal that binding affinities greater than ≈10^8^ M^−1^ (*K*_d_ ≤ 10 nM) can not be reliably measured under these conditions, and are indicative of essentially stoichiometric binding as evidenced by a paucity of data points in the transition region. At 5-10-fold-higher concentration of protein, which is more typical of the SBP-Mn(II) measurements in the literature (see Table 1), a *K*_a_ > 10^7^ M^−1^ (*K*_d_ < 100 nM) will not be reliably measured unless a chelator competitor, e.g., citrate for Mn(II), is used to measure *K*_a_^Mn^ (Grossoehme and Giedroc, 2009; Gribenko et al., 2013).

Since Mn(II) can only enter the cytoplasm efficiently through Mn(II)-specific transporters, elucidation of the affinity of each for Mn(II) vs. noncognate Zn(II) and the rates at which Mn(II) is transported across the plasma membrane takes on added significance when considered in the context of the discovery of calprotectin (see below). This is also true from the perspective of fundamental inorganic chemistry since Mn(II) complexes will tend to be far less thermodynamically stable than “isostructural” Zn(II) complexes (Figure 1A) (Waldron and Robinson, 2009). Metal transport studies have been carried out on bacterial NRAMP1 homologs in *S. typhimurium* and *E. coli* and generally show half maximal transport rates at 0.1 to 1 μM total Mn(II) depending on the transporter (Kehres et al., 2000). For ABC transporters, the concentration of any metal that gives maximal rates of transport has not yet been measured to our knowledge; on the other hand, the Mn(II) and Zn(II) affinities of the component SBPs have been determined using chelator competition assays or via direct titration by isothermal titration calorimetry (ITC). We compile these values here (Table 1) with the caveat that in an ITC experiment the affinity (*K*_d_ or *K*_a_) is often too tight to measure at the protein concentrations required to make the measurement (Figure 5), despite the ability to obtain a reliable measure of the stoichiometry and the heat of binding (Δ*H*_cal_). As can be seen, determined *K*_d_^Mn^ values range from the low nM to several hundred nM, with some indication that Zn(II) may bind more weakly than Mn(II) (Desrosiers et al., 2007; McDevitt et al., 2011; Zheng et al., 2011; Gribenko et al., 2013). We note that the *K*_d_^Mn^ obtained for *S. aureus* MntC of 4.0 ± 0.3 nM (50 mM citrate, 150 mM NaCl, pH 6.0, 20°C) is robust since this value was extracted from a nonstoichiometric binding isotherm acquired in the presence of 50 mM citrate as a Mn(II) competitor chelator (Gribenko et al., 2013). It will be interesting to learn how *K*_d_^Mn^ corresponds to *K*_m_ for transport, since rapid dissociation of Mn(II) from the SBP into the transport cavity (Pinkett et al., 2007) upon productive association with the transmembrane domain of the transporter (see Figure 4A) could facilitate rapid movement of Mn(II) across the membrane not fully reflected in the Mn(II)-binding thermodynamics (Figure 5).

**Table 1:** **Equilibrium dissociation constants (*K*_d_) reported for solute binding proteins of Mn-specific ATP-binding cassette transporters and for human calprotectin**.

**Protein**	**Method**	****K**_d_ (Mn), nM**	****K**_d_ (Zn), nM**
*Spn* PsaA SBP^a^	ITC	3.3 (±0.1)	230 (±2)
*Tpa* TroA SBP^b^	ITC	7.1 (±0.5)	22 (±4)
*Ype* YfeA SBP^c^	ITC	18 (±6)	6.7 (±1.6)
*Ssu* TroA SBP^d^	ITC	250 (±20)	430 (±10)
*Sau* MntC SBP^e^	ITC, competition	3.8 (±1.1)	not determined
Calprotectin + Ca(II)^f^,^g^	chelator comp	190; 21000	<0.01; <0.24
Calprotectin + Ca(II)^h^	ITC	1.3; 3700	1.4; 5.5

a 20 mM NaH_2_PO_4_, pH 6.5, 25 °C, 4.5–20 μM PsaA (McDevitt et al., 2011).

b 100 mM sodium acetate, pH 6.5, 20 °C, 40 μM TroA (Desrosiers et al., 2007).

c 100 mM sodium acetate, pH 6.5, 20 °C, 42 μM YfeA (Desrosiers et al., 2010).

d 20 mM sodium acetate, pH 6.5 at 25 °C, 30 or 90 μM TroA (Zheng et al., 2011).

e 50 mM citrate, 150 mM NaCl, 10% glycerol, pH 6.0, 25 °C, 36–43 μM MntC (Gribenko et al., 2013).

f Mn parameters measured in 75 mM Hepes, 100 mM NaCl, 0.2 mM Ca(II), pH 7.5, 25 °C. Values for metal site 1 (S1; His_6_) and metal site 2 (S2; His_3_Asp) are shown as K_d_^Mn1^, K_d_^Mn2^, K_d_^Zn1^, and K_d_^Zn2^ (Figure 5).

g Zn parameters measured in 75 mM Hepes, 100 mM NaCl, 1 mM Ca(II), pH 7.5, room temperature.

h Measured in 20 mM Tris, 100 mM NaCl, 22.5 μM Ca(II), 5 mM β-mercaptoethanol, pH 7.5, 30 °C, 7.5 μM CP (Kehl-Fie et al., 2011). A ΔS1 mutant CP does not bind Mn tightly in contrast to the ΔS2 mutant, revealing the His_6_ S1 site is the high affinity site for Mn (Damo et al., 2013).

## Host sequestration of transition metal ions

The mammalian host is a reservoir that is potentially rich in essential nutrients, including transition metals that must be acquired by bacterial pathogens (Versieck, 1985). In many cases, the host limits critical micronutrients such as iron (Weinberg, 1974) through both intracellular and extracellular complexation in an effort to withhold these metals from the invading pathogen (Weinberg, 1975; Hood and Skaar, 2012). For example, lipocalin 2 (Lcn2; siderochalin) binds Fe(III)-enterochelin, carboxymycobactin and bacillibactin complexes in direct competition with the bacterium (*E. coli*, *Mycobacterium tuberculosis*, or *Bacillus anthracis* in this case) that secretes these siderophores to capture bioavailable Fe from the host (Flo et al., 2004; Holmes et al., 2005; Sia et al., 2013). This establishes a competition between host and pathogen for the same metals (Bachman et al., 2009), and consistent with this model, Lcn2 expression and secretion is greatly elevated at sites of infection (Flo et al., 2004), and knockout mice lacking these and other host defenses are more susceptible to bacterial infection (Flo et al., 2004; Corbin et al., 2008; Hammer and Skaar, 2012).

Accumulating evidence assembled over the last several years reveals that a similar competitive strategy is used by the host to restrict the availability of both zinc and manganese in response to bacterial infection (Corbin et al., 2008; Kehl-Fie and Skaar, 2010; Kehl-Fie et al., 2011). This occurs in one of several ways. Macrophages and neutrophils are known to engulf intracellular pathogens in order to isolate them into a phagosomal compartment from which essential metals Mn and Fe are depleted by efflux, while Cu is concentrated (Wagner et al., 2005; White et al., 2009; Osman et al., 2010; Achard et al., 2012; Botella et al., 2012). Natural resistance-associated macrophage protein 1 (NRAMP1) (Cellier et al., 2007) and related H+-coupled transporters are known to efflux Mn(II) and Fe(II) from intracellular compartments of macrophages (Forbes and Gros, 2001), and knockout mice lacking NRAMP1 are susceptible to more virulent bacterial infections relative to wild-type mice (Skamene et al., 1982).

**Figure 6:**
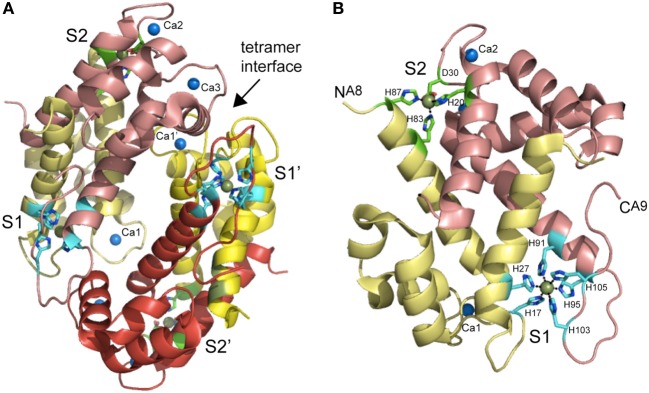
**Ribbon representation of the structure of the Mn(II)-bound CP heterotetramer (A) and S100A8-S100A9 heterodimer (B) (Damo et al., 2013)**. S100A8 chains are shaded in *yellow* and *pale yellow*, while S100A9 chains are shaded in *red* and *salmon*. The tetramer interface between the two heterodimers is marked. The two intersubunit Mn ions per heterodimer are shown as *smudge* spheres and the three Ca(II) ions per dimer are shown as *blue* spheres and labeled Ca1 bound to S100A8, Ca2 and Ca3, both bound to S100A9. The coordinating ligands to both S1 and S2 Mn(II) ions are shown in stick representation are labeled with residue number; one of the two S2 sites showed partial occupancy in the structure.

A number of S100 family proteins are now known to function extracellularly to chelate Mn(II) and Zn(II) to sequester these metals from the invading bacterium. For example, the S100A7 homodimer limits growth and invasion at epithelial surfaces through chelation of Zn(II) (Gläser et al., 2005), although the mechanistic details require further study. More recently it has been established that the heterotetrameric S100A8/S100A9 complex, also known as calprotectin (CP), binds both Mn(II) and Zn(II) (Figure 6) and is a major neutrophil-derived protein found in *Staphylococcus aureus*-induced tissue abscesses (Corbin et al., 2008). Laser ablation (LA)-ICP-MS was used to demonstrate that Mn(II) and Zn(II) were undetectable in abscesses relative to the surrounding uninfected tissue in a process dependent on host-encoded S100A8 and S100A9; furthermore, this chelation strategy is synergistic with neutrophil-mediated processes that sensitize these bacteria to superoxide stress by diminishing the effectiveness of Mn-SOD-dependent and independent antioxidant mechanisms (Kehl-Fie et al., 2011). These data support a model in which CP cripples bacterial defenses to both macrophage and neutrophil-mediated killing, and limits proliferation in tissue abscesses through Mn(II) chelation. More recent findings suggest that this general chelation strategy is likely operative in other tissues but is CP-independent, revealing that calprotectin may not be the only route that the host can use to limit Mn(II) from invading bacteria (Kehl-Fie et al., 2013). In addition, the degree to which Zn(II) chelation, relative to Mn(II), by CP limits bacterial growth is not fully understood, although a recent unbiased mutant screen carried out with the Gram-negative opportunistic respiratory pathogen *Acinetobacter baumannii* specifically identified components of the zinc acquisition and metabolism systems in that organism when challenged with CP (Hood et al., 2012; Moore et al., 2013).

## Calprotectin: structural, metal binding and functional properties

Recent studies reveal that CP has widespread antimicrobial activity against many Gram-positive and Gram–negative human pathogens grown in liquid culture, albeit to widely varying degrees, the explanation for which remains incompletely understood (Damo et al., 2013). We have recently shown that CP inhibits the growth of both wild-type and encapsulated strains of *Streptococcus pneumoniae* D39, extending the range of this broad spectrum antimicrobial activity (Lisher et al., unpublished). One simple explanation is that more resistant bacteria express Mn(II) uptake systems that possess a *higher* affinity than CP for Mn(II) and thus will compete more effectively with CP for extracellular Mn(II). This requires knowledge of the structure and Mn(II) and Zn(II) binding affinities of CP. Initial studies established that a single S100A8/S100A9 heterodimer is capable of binding two molar equivalents of transition metal, designated S1 and S2; however, while Zn(II) could fill both sites, Mn(II) could fill only one with high affinity (S1) (Kehl-Fie et al., 2011). The same is true in the context of the heterotetramer (S100A8_2_-S100A9_2_) which is well-modeled in metal binding experiments as two functionally independent heterodimers (Figure 6). Estimates of the metal binding affinity obtained in the presence of calcium from ITC show essentially stoichiometric binding at pH 7.5 with reported values of *K*_d_^Zn1^ = 1.4 nM and *K*_d_^Zn2^ = 5.6 nM (Table 1). For Mn(II), reported values are *K*_d_^Mn1^ = 1.3 nM and a *K*_d_^Mn2^ = 3.7 μM (Table 1). For reasons discussed above, nM values measured by ITC may well-reflect lower limits of *K*_a_ and correspondingly upper limits on *K*_d_ (see Figure 5). Indeed, subsequent metal competition experiments that employed a fluorescent sensor ZP4 (*K*_Zn_ = 0.65 nM; measurable range in *K*_Zn_ of 0.01–10 nM) as a competitor ligand for Zn(II) revealed *K*_d_^Zn1^ = 0.13 nM and *K*_d_^Zn2^ = 185 nM (–Ca) and *K*_d_^Zn1^ ≤ 0.01 nM and *K*_d_^Zn2^ ≤ 0.24 nM (+Ca) (Brophy et al., 2012), both significantly tighter than estimates from ITC. Room-temperature Mn(II) EPR titrations reveal *K*_d_^Mn1^ of 4.9 μM and *K*_d_^Mn2^ = 1.0 mM in the absence of calcium (Hayden et al., 2013) which shift to ≈200 nM and 21 μM in the presence of calcium (Table 1). These studies taken collectively reveal that calcium binding “switches on” CP to become a high affinity Zn/Mn binding protein with the major antimicrobial form of CP likely the mixed Mn (S1)/Zn (S2) heterotetramer (Hayden et al., 2013).

Crystallographic and mutagenesis experiments establish an unprecedented hexahistidine-coordinated (His_6_) Mn(II) site conforming to octahedral coordination geometry (Hayden et al., 2013) as the high affinity (S1) Mn(II) site, with the Zn(II) site (S2) adopting a tetrahedral His_3_-Asp complex (Damo et al., 2013) (Figure 6B). Two of the six histidines in the His_6_ site are derived from the conserved His103 and His105 in the C-terminal tail of S100A9 both of which are essential for antimicrobial function (Damo et al., 2013). The close proximity of these tail ligands to one of the Ca(II) binding sites immediately explains the strong calcium-dependent increase in Mn(II) binding affinity (Brophy et al., 2012). The broad-spectrum antimicrobial activity CP is largely dependent on the integrity of this S1 His_6_ site rather than the S2 Zn(II) site, thus likely connecting Mn(II) sequestration to the biological activity of CP. Although CP may have a lower affinity for Mn(II) than bacterially encoded SBPs (Table 1), the degree to which Mn(II) is coordinated by CP vs. SBPs or other Mn(II) import transporters is of course dictated by mass action which is set by the relative effective concentrations of each “chelator” in the milieu. Subsequent findings from Skaar and coworkers are consistent with this direct competition model, in that CP-imposed Mn(II) starvation increases the expression of a constitutively expressed NRAMP1-like manganese transporter, MntH, as well as the ABC family importer, MntABC, of which MntC is the Mn(II)-binding subunit (Kehl-Fie et al., 2013) (see Figure 4B). Both uptake systems are required to fully resist the effects of CP-dependent metal limitation since the IC_50_ for CP of an Δ*mntC*/Δ*mntH* stain was ≈50% lower than a *S. aureus* wild-type strain; additionally, both importers were required for resistance to superoxide stress as a result of increased SOD activity, and the ability of *S. aureus* to establish a systemic infection (Kehl-Fie et al., 2013). This “tug-of war” used to tip the balance of mass action in favor of the pathogen relative to Mn(II) acquisition may well be a general one (Champion et al., 2011).

## The impact of other transition metals on manganese acquisition and homeostasis

In addition to competition from host proteins of the innate immune response, there is some evidence that Zn(II) and Fe(II) can influence Mn(II) acquisition and intracellular Mn(II)-dependent metalloregulation of transcription. For example, Zn(II) has been shown to inhibit Mn(II) uptake by binding irreversibly to *S. pneumoniae* PsaA and effectively blocks Mn(II) transport into the cytoplasm (McDevitt et al., 2011). This induces an intracellular Mn(II) deficiency leading to upregulation of the expression of the entire PsaR regulon, as part of an effort to scavenge Mn(II) from the environment (Kloosterman et al., 2008; Jacobsen et al., 2011). In addition, Zn(II) is capable of binding to the Mn(II) sensor PsaR, converting PsaR into a poorly active repressor (Lisher et al., 2013) thereby minimizing transcriptional repression of *psaBCA* under these conditions (Jacobsen et al., 2011). Both of these effects can be reversed by the addition of Mn(II) to the growth media, suggesting the possibility that the pneumococcus may use one or both mechanisms to maintain a favorable intracellular Zn(II):Mn(II) ratio under conditions of high extracellular zinc toxicity that might occur in the lung, for example (McDevitt et al., 2011). Like PsaR (Lisher et al., 2013), other structurally related Mn(II)-specific metalloregulatory proteins, e.g., *B. subtilis* MntR, also bind Zn(II) with significantly higher affinity (≥100-fold), a finding consistent with the Irving-Williams series (Figure 1A) (Golynskiy et al., 2006; Maret, 2010; Ma et al., 2012), yet the Zn(II)-bound repressor binds much more weakly to the DNA operator than the cognate Mn(II)-bound repressor (Lieser et al., 2003). The degree to which this competition in the cell is a general strategy to modulate Mn(II) homeostasis under conditions of zinc toxicity is unknown.

Intracellular crosstalk between Fe(II) and Mn(II) homeostasis systems may well be more relevant to bacterial cell physiology and pathogenesis than is Zn(II)-Mn(II) crosstalk. These two metals lie at the same weakly competitive end of the Irving-Williams series and the Fe:Mn ratio might be considered a reporter of microbial lifestyle, capable of altering the altering the resistance of an organism to ROS (see Figure 1). As discussed above, a LMW-Mn(II) pool may well be present is most bacterial cells, albeit to differing degrees (Sharma et al., 2013), and some cells contain a chelatable pool of several hundred micromolar Fe(II) that is detectable by EPR spectroscopy of whole cells (Pericone et al., 2003). Thus, changes in the Mn:Fe ratio by upregulation of the Mn(II) acquisition system (Figure 2), or by crippling Fe(II) uptake repression in a *fur* mutant, for example, might be expected to change the metal specificity of an Fe(II)- or Mn(II)-specific metal-sensing repressor (Guedon and Helmann, 2003; Ma et al., 2011, 2012) and selected metalloenzymes (Whittaker, 2003; Anjem et al., 2009). This kind of Mn(II)-Fe(II) regulatory crosstalk is exemplified by recent work in *B. subtilis* on Mn(II)-MntR and two related Fur family members, the peroxide sensing, Fe(II)-binding PerR (see Figure 2) and the Fe(II)-sensing repressor Fur (Ma et al., 2011, 2012). It was reported that selected mutations of PerR introduced into a nonfunctional metal site (found in other Fur family proteins) close to the primary Fe(II) binding site altered the structure of PerR such that Mn(II) bound more tightly then Fe(II). Strains harboring these mutations were correspondingly more sensitive to peroxide stress since Mn(II)-PerR thus formed is unable to perform peroxide-catalyzed autooxidation which drives transcriptional derepression of the *perR* regulon. Remarkably, Fe-PerR-dependent H_2_O_2_-sensing was restored in this mutant in a *fur* mutant background, presumably allowing Fe(II) levels to rise to a level sufficient level to fill the mutant PerR regulatory site with cognate Fe(II) (Ma et al., 2011).

A second example concerns *B. subtilis* Fur itself (Ma et al., 2012). In this study, it was shown that cognate Fe(II) and noncognate metals Mn(II) and Zn(II) are equally effective in activating Fur to bind to its DNA operator *in vitro*; however, Fur is Fe(II)-specific *in vivo*. Remarkably, this Fe(II)-specificity is lost in a *perR* mutant strain. Here, the combined impact of increasing Fur concentrations and intracellular Mn(II) levels relative to Fe(II), leads to conditions where Fur binds Mn(II), which in turn, leads to inappropriate Mn(II)-mediated repression of the *fur* regulon, including genes responsible for Fe(II) uptake. Thus, PerR may directly impact Fe(II) homeostasis by modulating Fur levels in response to a change in the Mn:Fe ratio, i.e., that which might occur under conditions of high Mn(II) and Fe(II)-deplete conditions (see Figure 2).

Although the degree to which Fe(II)-Mn(II) crosstalk influences bacterial pathogenesis is not firmly established, a number of recent studies suggest that maintenance of an optimal Mn(II):Fe(II) ratio can impact the virulence of pathogenic bacteria. For example, the ability of *S. pneumoniae* to maintain a high Mn(II):Fe(II) ratio (Jacobsen et al., 2011) (Figure 1B) may be relevant to resistance to oxidative stress important for pathogenesis of this organism (Ong et al., 2013). In *Yersinia pestis*, the manganese import systems Yfe and MntH are regulated by Fe-Fur and loss of these systems leads to reduced virulence in sepsis models (Perry et al., 2012). In *Neisseria meningitidis*, a novel efflux protein, MntX, that maintains optimal Mn(II):Fe(II) ratios under conditions of low iron is also critical to virulence in sepsis models (Veyrier et al., 2011); this ensures that Fe(II) will remain bioavailable following conditions of high Mn(II) import in response to oxidative stress (see Figure 2). Interestingly, *Streptococcus pneumoniae*, like *Neisseria meningitidis*, also encodes a Mn(II) effluxer, MntE, that is required for virulence (Rosch et al., 2009). It will be interesting to see if the presence of a dedicated manganese effluxer represents a general strategy of providing a “release valve” to avert the effects of high intracellular Mn(II) particularly in Mn-centric bacterial pathogens (see Figure 1B).

## Concluding remarks

Recent studies of bacterial transition metal physiology and crosstalk places manganese acquisition by human microbial pathogens on center stage of the host-pathogen “arms race” (Botella et al., 2012). Mn(II) functions to metallate key enzymes, notably Mn-SOD that are responsible for the long-appreciated antioxidant properties of this metal. Recent insights from the application of sophisticated spectroscopies capable of probing Mn(II)-speciation in whole cells (Sharma et al., 2013), coupled with chemical investigations, provide strong support for the proposal that specific LMW-Mn complexes are catalytically competent and functionally important in clearing superoxide from cells, in a way that supplements SOD-dependent mechanisms (Barnese et al., 2012). The discovery of host immune proteins that limit biologically available Mn(II) for both intracellular and extracellular pathogens in an effort to cripple the resistance of invading pathogens to ROS establishes this as a general strategy used by the host to curtail bacterial infections (Corbin et al., 2008; Damo et al., 2013; Kehl-Fie et al., 2013).

Although structural and biophysical studies provide general support for a simple competition model in which the extracellular chelator calprotectin and Mn(II)-specific uptake systems compete for the same metal on the basis of their respective affinities, there is much more to be learned about this process. This includes elucidation of the rates and rate-limiting steps of Mn(II) transport, and structural studies of intact bacterial ABC transporters positioned at the “front line” of Mn(II) acquisition. This is particularly interesting since Mn(II) is generally handicapped relative to other divalent metal ions, notably Zn(II), in chelate stability, and as a result, other factors including formation of kinetically trapped Mn(II) metalloenzyme complexes in the cell (Whittaker, 2003; Tottey et al., 2008), may well be operative. In this context, it is interesting to note the *Streptococcus pneumoniae* expresses polyhistidine triad proteins (Pht) proteins attached on the cell surface known to bind zinc (Riboldi-Tunnicliffe et al., 2005) and thus could be used to scavenge Zn(II) from the host milieu under conditions of zinc limitation (Reyes-Caballero et al., 2010; Shafeeq et al., 2011; Plumptre et al., 2012, 2013). A bonus role for these proteins is that they could be used sequester Zn(II) and thereby reduce competition at the manganese importer PsaBCA, allowing pneumococcus to efficiently obtain Mn(II) which is likely bioavailable at vanishingly small quantities relative to Zn(II) (Shafeeq et al., 2013).

Further insights into molecular mechanisms of host nutritional immunity against bacterial pathogens will continue to rely on concerted and collaborative efforts of microbiologists, coordination chemists and structural biologists in an effort to win the “tug-of-war” over transition metals at the host-pathogen interface through the development of intervention strategies based on metals in biology of infectious disease.

## Author contributions

John P. Lisher and David P. Giedroc wrote the manuscript.

### Conflict of interest statement

The authors declare that the research was conducted in the absence of any commercial or financial relationships that could be construed as a potential conflict of interest.
